# Prechemoradiotherapy Systemic Inflammation Response Index Stratifies Stage IIIB/C Non-Small-Cell Lung Cancer Patients into Three Prognostic Groups: A Propensity Score-Matching Analysis

**DOI:** 10.1155/2021/6688138

**Published:** 2021-01-23

**Authors:** Erkan Topkan, Ugur Selek, Ahmet Kucuk, Veysel Haksoyler, Yurday Ozdemir, Duygu Sezen, Huseyin Mertsoylu, Ali Ayberk Besen, Yasemin Bolukbasi, Ozgur Ozyilkan, Berrin Pehlivan

**Affiliations:** ^1^Baskent University Medical Faculty, Department of Radiation Oncology, Baskent University, Adana, Turkey; ^2^Koc University Faculty of Medicine, Department of Radiation Oncology, Koc University, Istanbul, Turkey; ^3^Division of Radiation Oncology, The University of Texas MD Anderson Cancer Center, Houston, Texas, USA; ^4^Mersin City Hospital, Radiation Oncology Clinics, Mersin, Turkey; ^5^Medline Hospital, Clinics of Medical Oncology, Adana, Turkey; ^6^Baskent University Medical Faculty, Department of Medical Oncology, Baskent University, Adana, Turkey; ^7^Department of Radiation Oncology, Bahcesehir University, Istanbul, Turkey

## Abstract

**Purpose:**

We explored the prognostic influence of the systemic inflammation response index (SIRI) on the survival outcomes of stage IIIB/C non-small-cell lung cancer (NSCLC) patients who underwent concurrent chemoradiotherapy.

**Methods:**

Present propensity score-matching (PSM) analysis comprised 876 stage IIIB/C NSCLC patients who received 1–3 cycles of platinum-based doublets concurrent with thoracic radiotherapy from 2007 to 2017. The primary and secondary objectives were the relationships between the SIRI values and overall (OS) and progression-free survival, respectively. Propensity scores were calculated for SIRI groups to adjust for confounders and to facilitate well-balanced comparability between the SIRI groups by creating 1 : 1 matched study groups.

**Results:**

The receiver operating characteristic curve analysis identified an optimal SIRI cutoff at 1.9 for OS (AUC: 78.8%; sensitivity: 73.7%; specificity: 70.7%) and PFS (AUC: 80.5%; sensitivity: 75.8%; specificity: 72.9%) and we grouped the patients into two PSM cohorts: SIRI < 1.9 (*N* = 304) and SIRI ≥ 1.9 (*N* = 304), respectively. The SIRI ≥ 1.9 cohort had significantly worse median OS (*P* < 0.001) and PFS (*P* < 0.001) than their SIRI < 1.9 companions. The further combination of SIRI with disease stage exhibited that the SIRI-1 (IIIB and SIRI < 1.9) and SIRI-3 (IIIC and SIRI ≥ 1.9) cohorts had the best and worst outcomes, respectively, with SIRI-2 cohort (IIIB and SIRI ≥ 1.9 or IIIC and SIRI < 1.9) being remained in between (*P* < 0.001 for OS and PFS, separately). In multivariate analysis, the two- and three-laddered stratifications per the 1.9 cutoffs and SIRI groups retained their independent significance, individually.

**Conclusions:**

The SIRI ≥ 1.9 independently prognosticated significantly worse OS and PFS results and plated the stage IIIB/C patients into three fundamentally distinct prognostic groups.

## 1. Introduction

Results of two benchmark phase three randomized clinical trials set the concurrent chemoradiotherapy (C-CRT) as the current standard treatment choice for medically fit stage IIIB/C non-small-cell lung cancer (NSCLC) patients [1, 2]. Nevertheless, even with aggressive C-CRT, the outcomes of such patients remain grim with reported 5-year overall survival (OS) rates of only 15–20% [3]. Further efforts, including the escalation of radiotherapy (RT) dose from 60 to 74 Gy, also failed to improve these outcomes [4].

At present, the use of *T* and N components of the TNM (tumor-node-metastasis) staging system represents the best quality level strategy for choice of best-fit treatment and forecast of the outcomes of stage IIIB/C non-small-cell lung cancer (NSCLC) patients. However, the striking contrasts between the clinical outcomes of patients in comparative disease stages, even with the indistinguishable chemoradiotherapy (C-CRT) regimens, render the prognostic and predictive powers of the TNM framework flawed. Such significant outcome differences might be associated with the strict and sole reliance of the TNM system mainly on the size and local and regional extensions of the primary tumor, with no respect to the tumor- and host-related biological response factors, which may vary extensively among patients [5]. Consequently, the identification of novel biomarkers may serve usefully in more sophisticated prognostic stratification of such patients when utilized as an adjunct to the TNM staging system.

Systemic inflammation exhibits crucial roles in the promotion of all steps of the carcinogenic process from the initial cellular malignant transformation to the emergence of highly metastatic phenotypes [6, 7]. Hence, rationally, inflammatory blood constituents including the neutrophils (N), lymphocytes (L), monocytes (M), platelets, albumin, C-reactive protein, and cytokines/chemokines have been investigated in various blend forms for their predictive/prognostic essentialness in locally advanced NSCLCs [8–12]. In 2015, Qi et al. proposed a novel index to predict the survival of pancreatic cancer patients after chemotherapy, in particular the “systemic inflammation response index (SIRI)”, which was defined as SIRI = *N* × M/L [13]. The multivariate results of this study solidly confirmed the SIRI as an independent prognosticator for both the time to progression (TTP) and overall survival (OS). Subsequently, SIRI was shown to be also associated with clinical outcomes of metastatic pancreatic [14], renal [15], hepatocellular [16], nasopharyngeal [17], and esophageal cancers [18].

To our best information, despite the accessibility of strong evidence at other tumor sites, SIRI has never been assessed for its prognostic utility in stage IIIB/C NSCLC patients treated with radical C-CRT. Hence, we undertook this propensity score-matching (PSM) analysis in 876 stage IIIB/C NSCLC patients who received C-CRT to explore the prognostic influence of SIRI on their survival outcomes.

## 2. Materials and Methods

### 2.1. Patients

We retrospectively searched our institutional database to identify stage IIIB/C (AJCC 8th ed.) patients who underwent C-CRT with ≥1 chemotherapy cycle(s) between January 2007 and June 2017. Inclusion criteria were as follows: histopathologic documentation for NSCLC, stage IIIB/C disease by 18F-fluorodeoxyglucose positron emission tomography computerized tomography (PET-CT) according to AJCC 8th ed., age ≥18 and < 80 years, Karnofsky performance score (KPS) of 70–100, available pre-C-CRT brain magnetic resonance imaging (MRI) scans and detailed treatment records, and computerized treatment data sets. Patients with proven presence of malignant pleural/pericardial effusion, involved contralateral supraclavicular lymph nodes, inadequate pulmonary, cardiac, renal, or hepatic functions, and prior history of RT/chemotherapy were excluded.

### 2.2. Concurrent Chemoradiotherapy

All patients underwent PET-CT fusion-based RT planning as indicated by our institutional standards for locally advanced NSCLC patients and were treated with 3-dimensional conformal RT (3D-CRT) or intensity-modulated RT (IMRT) using megavoltage linear accelerators. The RT techniques, target volume definition, dose specifications, and normal tissue tolerance limits were as previously described by Topkan et al., elsewhere [19]. In brief, each patient received a total dose of 66 Gy thoracic RT in 33 fractions. All patients received 1–3 cycles of cisplatin/carboplatin plus one of docetaxel/paclitaxel (taxanes), vinorelbine, or etoposide during the C-CRT.

### 2.3. Measurement of SIRI

For each patient, the SIRI was calculated by utilizing the total blood count tests obtained on the first day of C-CRT with using Qi's original SIRI formula [13]: SIRI = *N* × M/L.

### 2.4. Response Assessment

After the C-CRT, patients underwent periodic response evaluations at 3-monthly (first two years), 6-monthly (third to fifth years), and yearly intervals thenceforth, utilizing the PERCIST criteria. Patient evaluations incorporated the blood count/chemistry and PET-CT, or chest CT scans (after confirmed complete response on PET-CT). Additional restaging tools were also utilized if indicated.

### 2.5. Statistics

Our primary objective was to evaluate the probable link between the SIRI and OS (interval between the first day of C-CRT and death/last visit). The secondary objective was the progression-free survival (PFS: interval between the first day of the C-CRT and the date of any type of disease progression/last visit/death). Quantitative variables were analyzed by using medians and ranges, while frequency distributions were used to describe categorical variables. Chi-square test, Student's *t*-test, Pearson's exact test, or Spearman's correlation estimates were used to compare frequency distributions, as indicated. Accessibility of pretreatment SIRI cutoff(s) that may stratify the study population into two gatherings with distinctive OS and PFS results was evaluated by using receiver operating characteristic (ROC) curve analysis. Owing to the fact that our current investigation was a retrospective cohort analysis, we calculated propensity scores for SIRI groups in order to adjust for confounding variables and to facilitate well-balanced comparability between the SIRI groups. For this purpose, we created 1 : 1 matched groups (nearest neighbor matching with logistic regression, caliper 0.2 without replacement) using the covariates age, sex, Karnofsky score, tumor histology, tumor stage, and cycles of concurrent chemotherapy. Patients were categorized into two or more groups for comparative analysis when necessitated, and respective Kaplan–Meier and log-rank tests were utilized to compare the possible risk factors for survival results. The Cox proportional-hazards model was applied for the multivariate analysis by including only the variables exhibiting significance in univariate analysis. All *P* values were 2-tailed and were considered significant if <0.05. Bonferroni correction was used to limit the haphazard false-positive results for simultaneously performed multiple subgroup analyses, such as the conceivable interactions between the tumor stages and SIRI groups.

### 2.6. Ethical Statement

The study design was approved by the institutional review board before collection of any patient data, and written informed consent was provided by each participant either themselves or legally authorized representatives for collection and analysis of blood samples, pathologic specimens, and publication of their outcomes.

## 3. Results

We identified a total of 876 stage IIIB/C NSCLC patients eligible for this analysis, with the patients and treatment baseline characteristics presented in Table 1. According to the PSM 1 : 1 shown in Table 1, 608 patients out of 876 were group-matched with 304 patients on each SIRI arm (≥1.9 versus <1.9). Patient characteristics remained homogeneously distributed on both groups following PSM (Table 1). All the following presented data represent the outcomes of PSM cohorts.

During this final analysis, 213 (35.0%) patients were still alive and 80 (13.2%) of them had no disease progression at a median follow-up time of 22.8 months (range: 2.7–156.3). For the whole PSM cohort, the median and 5-year OS rates were 24.5 months (95% confidence interval (CI): 23.2–25.8) and 19.6%, while the corresponding PFS rates were 10.9 months (95% CI: 10.1–11.7) and 11.4%, respectively.

**Figure 1:**
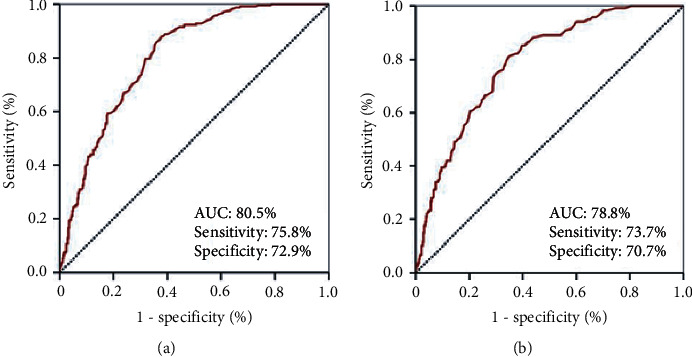
Results of receiver operating characteristic curve analyses: (a) progression-free survival; (b) overall survival.

**Figure 2:**
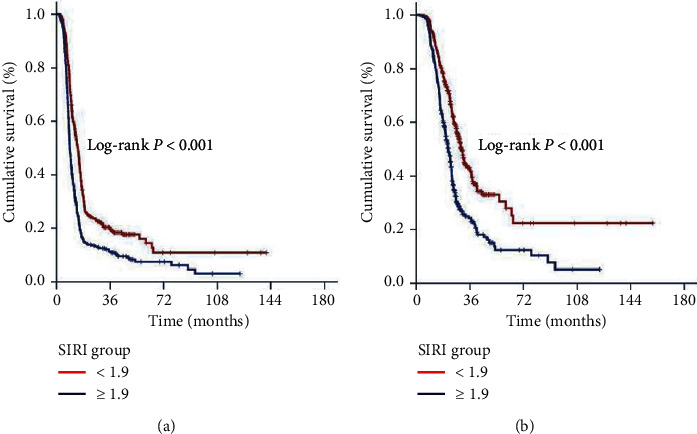
Comparative survival outcomes according to pretreatment SIRI groups (red line: SIRI < 1.9 and dark blue line: SIRI ≥ 1.9): (a) progression-free survival; (b) overall survival.

Search for a possible SIRI cutoff that may interact with treatment outcomes via utilizing ROC curve analysis revealed significance nearly at 1.9 for either of the OS (area under the curve (AUC): 78.8%; sensitivity: 73.7%; specificity: 70.7%) and PFS (AUC: 80.5%; sensitivity: 75.8%; specificity: 72.9%) as depicted in Figure 1. Grouping according to this cutoff revealed that the patients with SIRI <1.9 had significantly superior median OS (30.3 versus 21.3 months; *P* < 0.001), and PFS (14.2 versus 9.0 months; *P* < 0.001) durations than their counterparts with SIR I ≥ 1.9 (Figure 2). As given in Table 2, the actuarial 5- and 10-year OS and PFS outcomes also favored the SIRI <1.9 group, indicating that the outcomes were continuously worsening with a high SIRI value at long-term follow-up.

**Figure 3:**
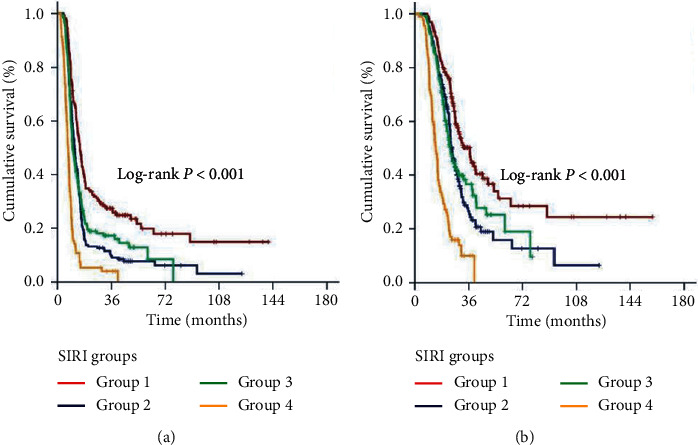
Comparative survival outcomes according to grouping composite disease stage and pretreatment SIRI groups (red line: Group 1, IIIB and SIRI < 1.9; dark blue line: Group 2, IIIB and SIRI ≥ 1.9; green line: Group 3, IIIC and SIRI < 1.9; and orange line: Group 4, IIIC and SIRI ≥ 1.9): (a) progression-free survival; (b) overall survival.

**Figure 4:**
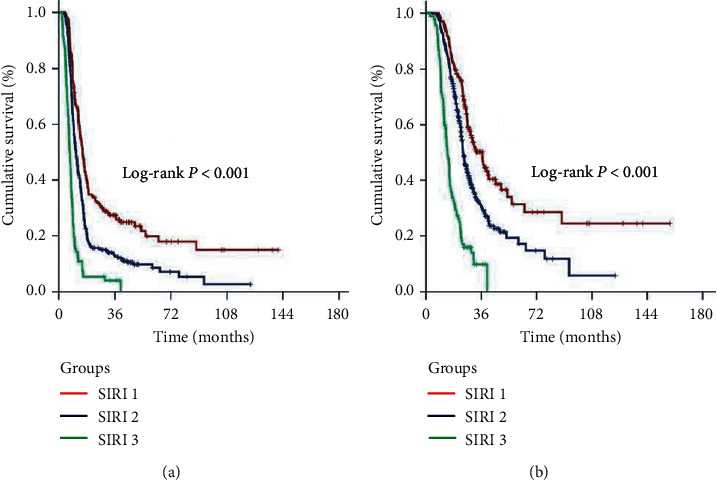
Comparative survival outcomes according to three-laddered pretreatment SIRI groups (red line: SIRI-1, IIIB and SIRI < 1.9; dark blue line: SIRI-2, IIIB and SIRI ≥ 1.9 or IIIC and SIRI < 1.9; and green line: SIRI-3, IIIC and SIRI ≥ 1.9): (a) progression-free survival; (b) overall survival.

In an effort to distinguish particular patients' groups with significantly distinct outcomes, we further grouped the patients according to disease stage and SIRI status, Group 1: stage IIIB + SIRI < 1.9; Group 2: stage IIIB + SIRI ≥ 1.9; Group 3: stage IIIC + SIRI < 1.9; and Group 4: stage IIIB + SIRI ≥ 1.9, respectively. Intergroup comparisons revealed that Group 1 patients had the best median survival outcomes (36.7 and 15.0 months for OS and PFS, respectively) while Group 4 patients exhibited the worst outcomes group (13.8 and 7.1 months for OS and PFS, respectively). In this respect, representing the intermediate outcome groups, the outcomes of Group 2 (24.6 and 11.4 months for OS and PFS, respectively) and Group 3 (23.5 and 10.2 months for OS and PFS, respectively) patients were statistically indistinguishable (Figure 3). One step further, we stratified the patients into 3 final outcome groups, SIRI-1: Group 1; SIRI-2: Group 2 or 3; and SIRI-3: Group 4 patients, respectively. Bonferroni corrected (*P* < 0.017 for significance) results of our final 3 laddered stratification revealed that the median and long-term survival differences between the 3 groups were statistically significant with all favoring the early SIRI groups (*P* < 0.001 for each of Group 1 versus 2, Group 1 versus 3, and Group 2 versus 3), as presented in Table 2 and Figure 4. Search for possible reasons for the extremely poor outcomes in the SIRI-3 group uniquely discovered that 69.7% of all distant metastases expressed themselves in the first 8 months of the post-C-CRT period in these patients' cohort.

Results of univariate analyses revealed that the early tumor stage (IIIB versus IIIC), higher KPS (90–100 versus 70–80), early T-stage (1–2 versus 3–4), lower N-stage (2 versus 3), lower SIRI (<1.9 versus ≥1.9), and earlier SIRI groups (SIRI-1 versus SIRI-2 versus SIRI-3) were the factors with the significantly superior OS and PFS outcomes (Table 3), and all of which retained their independent prognostic significance in multivariate Cox regression analysis (Table 3).

## 4. Discussion

The three main discoveries of present PSM analysis comprising 608 stage IIIB/C NSCLC patients were as follows: first, we demonstrated that the pre-C-CRT SIRI ≥ 1.9 was an independent predictor of poor prognosis concerning the OS (*P* < 0.001) and PFS (*P*=0.001). Second, SIRI was able to stratify the stage IIIB/C patients into 3 SIRI subgroups with fundamentally distinct prognosis independent of the TNM staging. And third, proposing urgent treatment revisions, the SIRI-3 patients exhibited extremely poor PFS and OS results reminiscent to the stage IVB NSCLC patients.

Granting its critical roles in the initiation and fatal progression ventures of carcinogenesis, inflammation has become the seventh hallmark of cancer [6]. Notwithstanding the traditionally perceived tumor-related factors, growing inflammation research proof has indicated that the prognosis of cancer patients was moreover firmly associated with the host-related factors, essentially the systemic inflammatory response which promotes tumor angiogenesis, suppresses antitumor immunity, facilitates escape from immune response, accelerates the metastatic process, and induces resistance to anticancer therapies including the chemotherapy and radiotherapy [20]. Previous studies have confirmed the prognostic utility of various systemic inflammation markers, such as N-to-L ratio (NLR), P-to-L ratio (PLR), and L-to-M ratio (LMR), prognostic nutritional index, Glasgow prognostic index, systemic immune-inflammation index, and advanced lung cancer inflammation index in NSCLC patients undergoing various treatments [4–8, 21]. Another recently emerged blood-based biomarker is the SIRI, which has been shown to reliably predict survival outcomes in various cancers [13–18]. Interestingly, although the background is strong enough, the prognostic value of SIRI has never been investigated in stage IIIB/C NSCLC patients treated with radical C-CRT, which formed a rational basis for this present first attempt PSM analysis evaluating the prognostic worth in this patients' group.

The first important finding of this PSM analysis was the first show of prognostic incentive for SIRI in the stratification of stage IIIB/C NSCLC patients into two significantly distinct prognostic groups with regards to the PFS and OS outcomes at a cutoff of 1.9, which appeared, by all accounts, to be independent of the traditional KPS, T-stage, N-stage, and TN-stage. Albeit, we cannot compare our results with other NSCLC research in the absence of similar studies, yet they are in good accordance with the published SIRI results in other tumor primaries [13, 18]. In the first SIRI study, SIRI was recommended as a novel indicator of longer TTP (*P*=0.003) and OS (*P* < 0.001) in advanced pancreatic cancer patients after chemotherapy [13], which was later confirmed by other successive pancreatic, renal, hepatocellular, nasopharyngeal, and esophageal cancer investigations [14, 18]. Though further investigations at other tumor sites are required, yet, the available proof reasonably suggests the SIRI as a common prognostic predictor of clinical outcomes for many tumor types, including the stage IIIB/C NSCLCs undergoing definitive C-CRT, as presented here.

The unique finding of our PSM analysis was the exhibition of the ability of SIRI in distinguishing stage IIIB/C patients into three subgroups with significantly distinct PFS and OS outcomes: SIRI-1 (IIIB and SIRI < 1.9), 15 and 36.7 months; SIRI-2 (IIIB and SIRI ≥ 1.9, or IIIC and SIRI < 1.9), 11.1 and 23.9 months; and SIRI-3 (IIIC and SIRI ≥ 1.9), 7.1 and 13.8 months, respectively. Though it needs to be confirmed by other series, we believe that the easy-to-calculate, replicable, objective, and free of financial burden SIRI is an independent prognostic marker, which is encouraging in the more confident selection and stratification of stage IIIB/C patients in running trials to increase the homogeneity of the study cohorts for expected survival, as well as for the increased relevancy of the results in any treatment comparisons.

We observed that the SIRI-3 (stage IIIC with SIRI ≥ 1.9) patients displayed a remarkably inferior median survival of 13.8 months with none being alive at 5 years compared to their SIRI-2 (stage IIIC with SIRI < 1.9) counterparts. This finding is of particular importance as it confirms the robust discriminative prognostic power of the SIRI in NSCLC patients coded to the same TNM stage. Furthermore, these outcomes in the SIRI-3 group are clinically alarming as they resemble the 3 to 11.5 month range reported for stage IVB NSCLC patients after traditional palliative chemotherapy [22]. Interpreting this particular observation together with a median PFS of only 7.1 months and the fact that 69.7% of all distant metastases were diagnosed in the first 8 months of the post-C-CRT period, our findings strongly propose that most patients in this particular group had already harbored occult metastatic foci before C-CRT that were far below the resolution capacity of accessible staging tools including the PET-CT. Present poorer PFS in SIRI-3 also calls for another reasonable explanation of this finding: inflammation-induced chemo- and radio-resistance of the primary tumor and fired systemic metastasis [11]. Whatever the exact reason is, such information might serve useful for more accurate stratification of stage IIIC NSCLC patients who require enhancement of their systemic treatments beyond that of the standard C-CRT until the implementation of more sophisticated tools to the routine NSCLC staging practice, such as the liquid biopsy procedures.

In the search for a more robust systemic treatment for SIRI-3 patients, who by outcomes resemble the stage IVB patients, our study cohort did not have the diversity to see the utility of induction chemotherapy. However, recently Yi et al. proposed the systemic inflammation marker NLR as a predictor of outcomes after first-line chemotherapy in advanced NSCLCs, which might simulate an induction chemotherapy cohort analyzed with a systemic inflammation marker [23]. The authors documented that the prechemotherapy NLR ≥2.16 was significantly associated with worse median OS, almost one-third of the median OS of NLR <2.16 cohort. Similarly, NLR ≥1.65 was noted to predict poorer tumor response after 2 cycles of chemotherapy. These particular findings logically propose that the NSCLCs presenting with high systemic inflammation might also be not good candidates for doing better even with intensive induction chemotherapies. In this setting, because the high SIRI patients may be presenting with unidentified occult distant metastases before C-CRT, the novel immunotherapeutic agents might be suggested as an alternative to enhance the systemic treatment. Recently, Liu et al. reported that the systemic immune-inflammation index, NLR, and PLR levels were linked with nivolumab efficacy in 44 advanced NSCLC patients; likewise, in the abovementioned chemotherapy study, the high-SII group had worse OS (*P*=0.005) and PFS compared with the low-SII group (*P*=0.006) even with immunotherapy [24], which was confirmed with the results of two meta-analyses supporting the association between a low pretreatment systemic inflammation and better outcomes for NSCLCs treated with immunotherapy [25, 26]. Furthermore, Ye et al. pointed out that any decrease in immune surveillance was contributing to the escape of circulating tumor cells from the immunocytes with resultant formation of new metastases [27]. Therefore, patients with high systemic inflammation scores, such as SIRI ≥1.9, might also be not responding well to immunotherapy on the background of strong correlations between the enhanced homing of circulating tumor cells in an inflammatory microenvironment and high incidences of distant metastases. To our understanding, all these results appear to be suggestive for the addition of highly efficient anti-inflammatory agents to the treatment algorithm of the SIRI-3 NSCLC patients. Confirming this suggestion, the results of a phase I study by Komaki et al. are encouraging [28]. In this study, the authors administered the cyclooxygenase-2 inhibitor celecoxib concurrent with the cisplatin and irinotecan doublet during the C-CRT course of unresectable locally advanced NSCLC patients. The results were highly impressive with 2-year 65% OS, 64% PFS, 69% locoregional control, and 71% distant-metastasis-free survival rates with no notable increments in the toxicity rates. This finding is hypothesis-generating for the possible use of celecoxib in unfavorable high SIRI groups to observe whether it may improve the expected poor survival outcomes or not.

The present study has some certain drawbacks. First, acknowledging the unpredictable biases of any single-institutional retrospective cohort analysis, we believe that our results ought to be interpreted as hypothesis-generating as opposed to a strong proposal. Second, the incorporation of NSCLCs treated with 1 to 3 cycles of concurrent chemotherapy might be condemned as a confounding factor on the outcomes reported herein. Nevertheless, such an approach is undoubtedly advantageous in evaluating a group of patients, which reflects the real-world routine practice rather than a highly selected population. Third, the debated use of consolidation chemotherapy in some patients and differences between the intensities and types of salvage chemotherapy regimens may likewise have altered the outcomes in favor of either SIRI groups. Fourth, albeit SIRI is a dynamic biomarker of immune and inflammation status, which may vary broadly during and post-C-CRT periods, yet present investigation concentrated solely on the pre-C-CRT SIRI values. Therefore, to define potentially more reliable cutoff(s), future research ought to focus on SIRI dynamics. Finally, despite our endeavors to reduce heterogeneity in our patient population by using the PSM analysis according to clinical and tumor variables, the robustness of present results ought to be confirmed by future large-scale PSM data sets.

## 5. Conclusions

Present results in our PSM cohort of 608 stage IIIB/C patients treated with radical C-CRT showed that the baseline SIRI ≥1.9 was an independent robust biomarker to predict significantly worse OS and PFS results and identified three SIRI subgroups in stage IIIB/C patients with flawless outcome stratification beyond that of conventional TNM staging alone. The particular finding of only 13.8 months of median OS duration in the SIRI-3 group, which was in the range of previously estimated median OS durations of stage IVB patients, strongly underlines the urgent need for implementation of more effective systemic agents to the treatment algorithm of such patients.

## Figures and Tables

**Table 1:** Pretreatment and treatment characteristics for all and PSM patients per SIRI status.

Covariate	All patients (*N* = 876)	SIRI ≥ 1.9 (*N* = 487)	SIRI < 1.9 (*N* = 389)	*P* value	PSM patients (*N* = 608)	SIRI ≥ 1.9 (*N* = 304)	SIRI < 1.9 (*N* = 304)	*P* value
Median age, *y* (range)	64 (29–79)	65 (31–79)	63 (29–79)	0.56	65 (31–79)	64 (31–79)	65 (34–79	0.87

*Age group, y* (%)								
≤70 years	772 (88.1)	428 (86.7)	344 (88.4)	0.64	542 (89.1)	270 (88.8)	272 (89.5)	0.82
>70 years	104 (11.9)	59(13.3)	45 (11.6)		66 (10.9)	34 (11.2)	32 (10.5)	

*Gender, n* (%)								
Male	539 (61.5)	291 (59.8)	248 (63.8)	0.32	368 (60.5)	180 (59.2)	188 (61.8)	0.49
Female	337 (38.5)	196 (40.2)	141 (36.2)		240 (39.5)	124 (40.8)	116 (38.2)	

*KPS, n* (%)								
90–100	751 (71.7)	269 (71.0)	482 (72.0)	0.64	442 (72.7)	219 (72.0)	223 (73.4)	0.79
70–80	297 (28.3)	110 (29.0)	187 (28.0)		166 (27.3)	85 (28.0)	81 (26.6)	

*Histology, n* (%)								
AC	567 (54.1)	212 (55.9)	355 (53.1)	0.39	326 (53.6)	157 (51.6)	169 (55.6)	0.32
SCC	481 (45.9)	167 (44.1)	314 (46.9)		282 (46.4)	147 (48.4)	135 (44.4)	

*T-stage, n* (%)								
1–2	361 (34.4)	116 (30.6)	245 (36.6)	0.28	254 (41.8)	130 (42.8)	124 (40.8)	0.55
3–4	687 (65.6)	263 (69.4)	424 (63.4)		354 58.2)	174 (57.2)	180 (59.2)	

*N-stage, n* (%)								
2	531 (50.7)	195 (51.5)	336 (50.2)	0.55	317 (52.1)	161 (53.0)	156 (51.3)	0.76
3	517 (49.3)	184 (48.5)	333 (49.8)		291 (47.9)	143 (47.0)	148 (48.7)	

*Tumor stage, n* (%)								
IIIB	617 (58.9)	217 (57.3)	400 (59.8)	0.67	369 (60.7)	188 (61.8)	181 (59.5)	064
IIIC	431 (41.1)	162 (42.7)	269 (40.2)		239 (39.3)	116 (38.2)	123 (40.5)	

*Abbreviations*. SIRI: systemic inflammation response index; PSM: propensity score matching; KPS: Karnofsky performance score; AC: adenocarcinoma; SCC: squamous cell carcinoma; T: tumor; N: node.

**Table 2:** Survival outcomes according to the systemic inflammation response index (SIRI) status and SIRI subgroups.

Outcome	SIRI < 1.9 (*N* = 304)	SIRI ≥ 1.9 (*N* = 304)	*P* value	SIRI-1 (*N* = 171)	SIRI-2 (*N* = 345)	SIRI-3 (*N* = 92)	^*∗*^ *P* value
*PFS*							
Median, mo	14.2 (12.8–15.6)	9.0 (8.4–9.6)	<0.001	15.0 (13.2–16.8)	11.1 (10.2–12.0)	7.1 (6.3–7.9)	<0.001
5 years (%)	7.4	16.0		19.8	9.8	0	
10 years (%)	3.1	10.8		14.9	NR	0	

OS							
Median, mo	30.3 (27.8–32.8)	21.3 (19.5–23.1)	<0.001	36.7 (30.6–42.8)	23.9 (22.3–25.5)	13.8 (12.2–15.4)	<0.001
5 years (%)	12.3	30.5		31.4	19.3	0	
10 years (%)	5.1	22.4		24.4	5.9	0	

*∗*Bonferoni corrected *P* value <0.017 for significance. *Note*. SIRI-1: stage IIIB and SIRI < 1.9; SIRI-2: stage IIIB and SIRI ≥ 1.9 or stage IIIC and SIRI < 1.9; SIRI-3: stage IIIC and SIRI ≥ 1.9. *Abbreviations*. SIRI: systemic inflammation response index; PFS: progression-free survival; OS: overall survival.

**Table 3:** Results of univariate and multivariate analyses.

Characteristic	Patients (N)	Median PFS (months)	Univariate *P* value	Multivariate *P* value	Median OS (months)	Univariate *P* value	Multivariate *P* value
*Age group*							
<70 years	542	11.3	0.28	—	25.0	0.17	—
≥70 years	66	10.1			23.1		

*Gender*							
Male	368	10.6	0.58	—	23.9	0.39	—
Female	240	11.5			25.8		

*KPS*							
90–100	442	12.3	0.001	0.002	27.4	0.0003	0.004
70–80	166	9.2			21.6		

*Histology*							
SCC	326	10.7	0.92	—	23.8	0.72	—
AC	282	11.1			24.9		

*T-stage*							
1–2	254	13.2	<0.001	<0.001	28.7	<0.001	<0.001
3–4	354	9.9			21.8		

*N-stage*							
2	317	13.2	<0.001	<0.001	29.3	<0.001	<0.001
3	291	9.6			20.2		

*Stage*							
IIIB	369	13.4	<0.001	<0.001	29.6	<0.001	<0.001
IIIC	239	9.6			21.9		

*SIRI*							
<1.9	304	14.2	<0.001	<0.001	30.3	<0.001	<0.001
≥1.9	304	9.0			21.3		

*SIRI group*							
1	171	15.0	<0.001	<0.001	36.7	<0.001	<0.001
2	345	11.1			23.9		
3	92	7.1			13.8		

*Note*. SIRI groups: SIRI-1, stage IIIB and SIRI < 1.9; SIRI-2, stage IIIB and SIRI ≥ 1.9 or stage IIIC and SIRI < 1.9; SIRI-3, stage IIIC and SIRI ≥ 1.9. *Abbreviations*. PFS: progression-free survival; OS: overall survival; KPS: Karnofsky performance score; SCC: squamous cell carcinoma; AC: adenocarcinoma; T: tumor; N: node; SIRI: systemic inflammation response index.

## Data Availability

The datasets used and/or analyzed during the current study are available from the Baskent University Department of Radiation Oncology Institutional Data Access for researchers who meet the criteria for access to confidential data: contact address, adanabaskent@baskent.edu.tr.
